# Causal associations of hand grip strength with bone mineral density and fracture risk: A mendelian randomization study

**DOI:** 10.3389/fendo.2022.1020750

**Published:** 2022-12-12

**Authors:** Jidong Song, Tun Liu, Jiaxin Zhao, Siyuan Wang, Xiaoqian Dang, Wei Wang

**Affiliations:** The Second Affiliated Hospital, Xi’an Jiaotong University, Xi’an, Shaanxi, China

**Keywords:** hand grip strength, sarcopenia, bone mineral density, fracture risk, Mendelian randomization

## Abstract

**Background:**

Muscle strength has been shown to exert positive effects on bone health. The causal relationship between hand grip strength and osteoporosis is an important public health issue but is not fully revealed. The goal of this study was to investigate whether and to what extent hand grip strength affects bone mineral density (BMD) and fracture risk.

**Methods:**

We conducted a state-of-the-art two-sample Mendelian randomization analysis. Genomewide significant (*P*<5×10^-8^) single nucleotide polymorphisms associated with hand grip strength were obtained. Summary level data of BMD and fractures at different body sites (lumbar spine, heel, forearm and femoral neck) was obtained from a large-scale osteoporosis database. The inverse variance weighted method was the primary method used for analysis, and the weighted-median, MR-Egger were utilized for sensitivity analyses.

**Results:**

The results provided strong evidence that hand grip strength trait was causally and positively associated with lumbar spine BMD (*β*: 0.288, 95% CI: 0.079 to 0.497; *P*=0.007), while no causal relationship was found between hand grip strength and BMD at heel (*β*: -0.081, 95% CI: -0.232 to 0.070; *P*=0.295), forearm (*β*: 0.-0.101, 95% CI: -0.451 to 0.248; *P*=0.571) or femoral neck (*β*: 0.054, 95% CI: -0.171 to 0.278; *P*=0.639). In addition, no statistically significant effects were observed for hand grip strength on fracture risks (*β*: -0.004, 95% CI: -0.019 to 0.012; *P*=0.662).

**Conclusions:**

This study showed a positive causal relationship between hand grip strength and lumbar BMD, which is the most common site of osteoporotic fracture, but did not find a causal relationship between hand grip strength and BMD of heel, forearm, or femoral neck. No statistically significant effect of hand grip strength on fracture risk was observed. This study indicates variations in the abilities of hand grip strength trait to causally influence BMD at different skeleton sites. These results should be considered in further studies and public health measures on osteoporosis prevention strategies.

## 1 Introduction

Osteoporosis is a common musculoskeletal disorder characterized by low bone mass and deterioration of bone microstructure, resulting in decreased bone density and increased risk of fracture. The incidence of osteoporosis increases significantly with age. The prevalence of osteoporosis is 16.0% among men aged 50 years or older and 29.9% among postmenopausal women, and the annual cost of osteoporotic fractures is estimated to reach $25 billion by 2025 in the USA (1–3). Low bone mineral density (BMD) and fracture risk are two major characteristics of osteoporosis. Although several genetic loci influencing this disease have been detected, the genetic mechanism is still not fully understood.

Sarcopenia is also an age-related condition characterized by progressive and generalized accelerated loss of muscle mass and function, associated with an increased likelihood of adverse outcomes including falls, functional decline, frailty, and mortality (4, 5). The stepwise diagnostic protocol starts with the measurement of muscle strength, including grip strength and chair stand tests (5, 6). Prevalence estimations for sarcopenia vary widely across clinical settings, with reported prevalence rates of 1-29% in community-dwelling residents and 14-33% in residents requiring long-term care (7, 8), resulting in an estimated $18.5 billion in direct medical costs in the USA in 2000 (9). Osteoporosis and sarcopenia may coexist in the elderly.Identifying the relationship between the two may have implications for clinicians to intervene and improve osteoporosis (10). The grip strength test is a simple and effective way of measuring muscle strength (11). However, the epidemiological conclusions on the relationship between grip strength and BMD or fracture risk remain inconsistent (12–14). Moreover, it is not clear whether these relationships are causal because of the inherent limitations of conventional observational studies, including small sample sizes and confounding and reverse causality. Although randomized controlled trials (RCTs) are the gold standard for inferring causality, they are expensive, time-consuming and sometimes impractical.

The popularity of genome-wide association analysis (GWAS) has revolutionized the study of human genetics and the genetic mechanisms of complex diseases (15). Mendelian randomization (MR) uses GWAS data to analyze the causal relationship between different exposures and outcomes. Alleles follow the law of independent assortment and are constant during their whole lifetime, which imitates the design of an RCT (16). MR analyses effectively overcome the limitations of traditional observational studies. Therefore, MR is a feasible way to analyze the causal association between grip strength and BMD or fracture risk.

Here, we performed two-sample MR analysis using large-scale GWAS summary statistics to explore the causal associations of BMD at different skeletal sites and the risk of bone fracture with grip strength.

## 2 Methods

### 2.1 Study design

**Figure 1:**
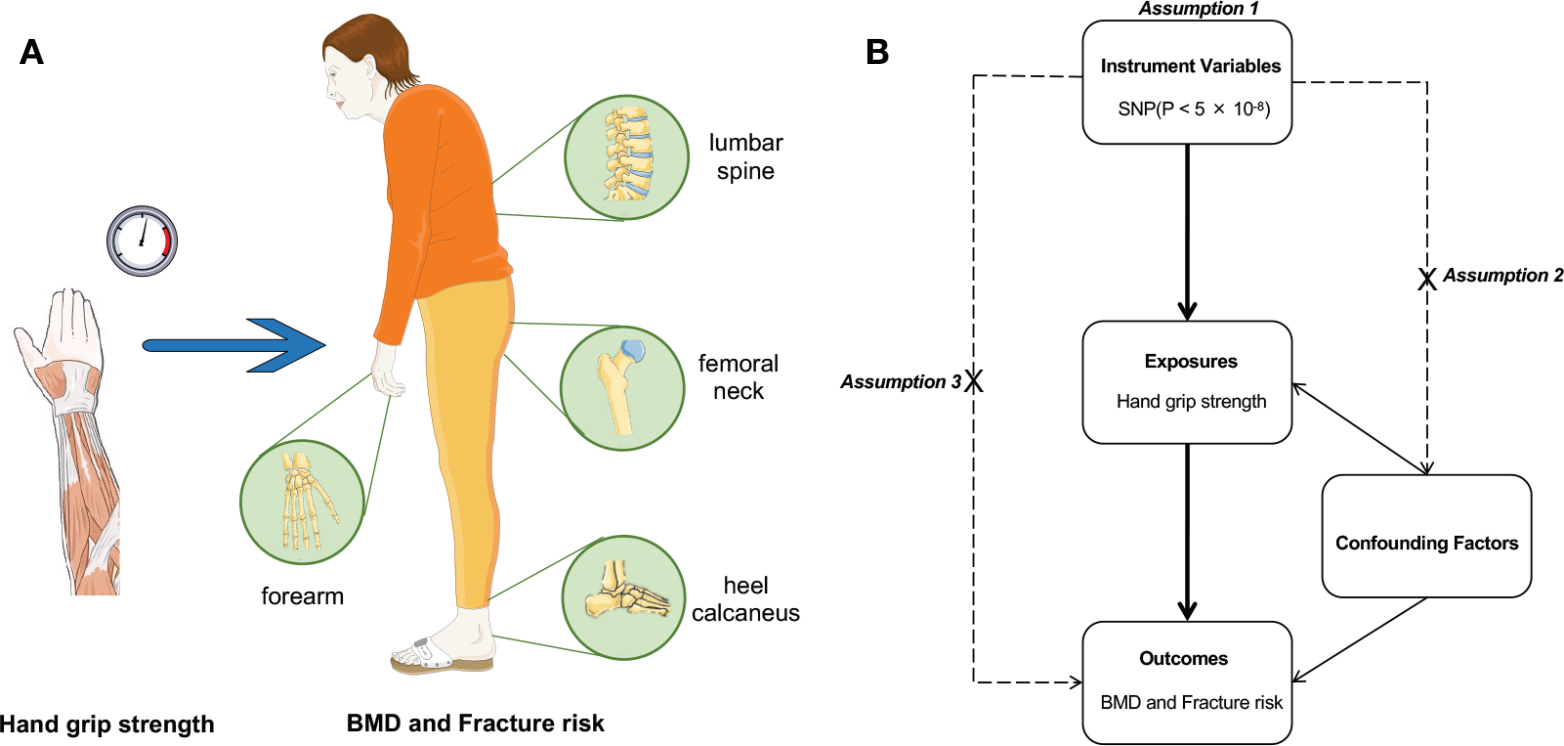
The study design of two‐sample MR analysis. **(A)** The schematic diagram of our study design. **(B)** Assumptions underlying a MR analysis. MR, Mendelian randomization.

Our study utilized a two-sample Mendelian randomization analysis of grip strength with different bone locations. Hand grip strength was categorized as the exposure, and BMD at four skeletal sites (heel, forearm, lumbar spine and femoral neck) and fracture risk were considered outcomes. MR is based on three main assumptions (15): the instrumental variables should be correlated with the exposure; the instrumental variables should not be associated with confounders; and the instrumental variables should influence the outcome only through the exposure (no horizontal pleiotropy) (**Figure 1**). The significant genome-wide single-nucleotide polymorphisms (SNPs) (*P*<5×10^-8^) were selected as instrumental variables. Further sensitivity and pleiotropy analyses were performed to ensure the robustness of the results.

### 2.2 Data sources

The participants of the GWAS are of European descent. For the exposure, the summary statistics data on hand grip strength (right) were retrieved from the United Kingdom Biobank (UKB), including 499,260 white British individuals.

For the outcomes, the summary statistics data on BMD of the femoral neck, lumbar spine and forearm were retrieved from the Genetic Factors for Osteoporosis Consortium (GeFOS), including 53,236 individuals (17). The datasets for the eBMD of the heel calcaneus and fracture risk were obtained from the UKB, including 142,487 participants (18).

### 2.3 Instrumental variable selection

To select instrumental variables that satisfy the three assumptions of the MR analysis, we performed the following five steps. Genome-wide single-nucleotide polymorphisms (SNPs) that are closely associated with hand grip strength were identified from the exposed GWAS (*P*<5×10^-8^). To estimate linkage disequilibrium (LD) between SNPs, a clumping process (r^2^>0.6, window size=250 kb or 1000 SNPs) was performed on 1000 Genomes Project data (19). For specific requested SNPs not present in the BMD GWAS, their LD proxies were estimated using 1000 Genomes Project data (19, 20). SNPs with minor allele frequencies <0.05 were further excluded. Ambiguous SNPs with nonconcordant alleles (e.g., G/A vs. G/T) were excluded, and coordinates with ambiguous palindromic SNPs were harmonized (e.g., A/T vs. C/G).

### 2.4 Statistical analyses

In this study, we performed an inverse variance weighted (IVW) meta-analysis to analyze each Wald ratio to initially estimate the causal relationship between exposure and outcome. However, if any evidence of horizontal pleiotropy exists in the IV, this method is considered biased in estimating causality, and the robustness of the IVW method depends on the pleiotropy of IV. Even when nearly 50% of SNPs are invalid instrumental variables, the weighted median method yields an estimate that is compatible with the final effect; this approach can be used to achieve unbiased estimates of causal effects in the presence of unbalanced level pleiotropy. Under the InSIDE assumption that instrumental variables are independent of direct effects, MR–Egger regression can provide consistent estimations even if all SNPs are not valid instrumental variables. Nevertheless, MR–Egger estimates are less accurate than weighted median methods and may be affected by outlying genetic variants. We also used MR–Egger regression intercepts to assess directional pleiotropy and ‘leave-one-out’ sensitivity analysis to evaluate whether causal effects were driven by a single potentially influential SNP. The association between exposure and outcome phenotype was considered statistically significant at *P*<0.05. All MR analyses were performed using the ‘TwoSampleMR’ package in R software.

## 3 Results

### 3.1 Casual relationships between hand grip strength and BMD

**Figure 2:**
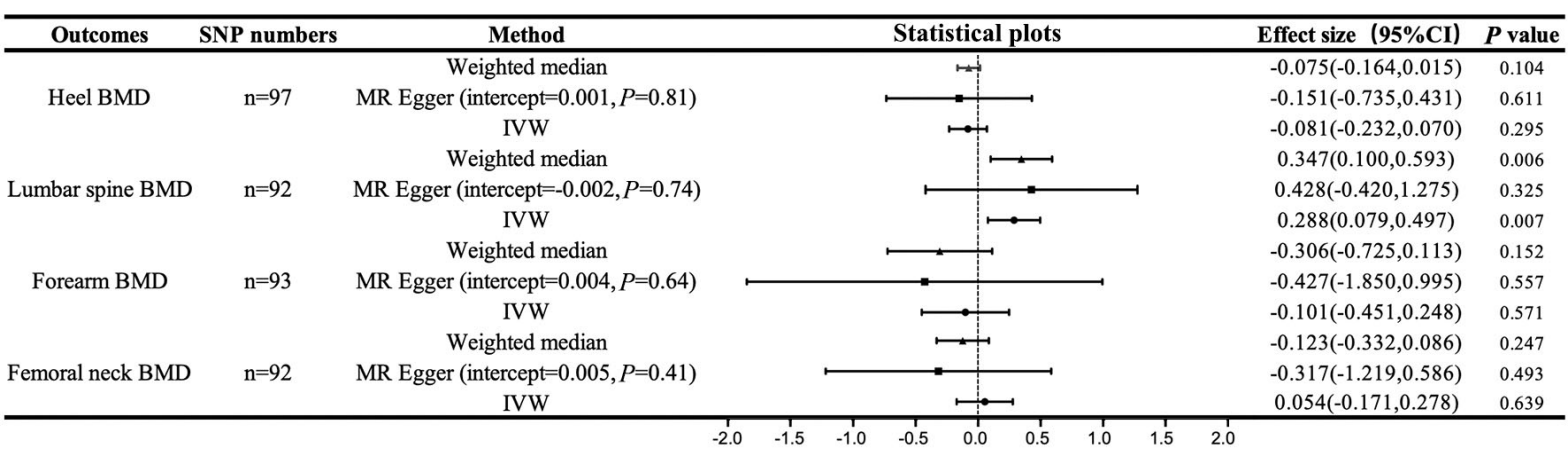
Casual associations between hand grip strength and BMD. IVW, inverse variance weighted; MR Egger, mendelian randomization egger; CI, confidence interval; BMD, bone mineral density; SNP, single nucleotide polymorphism.

**Figure 3:**
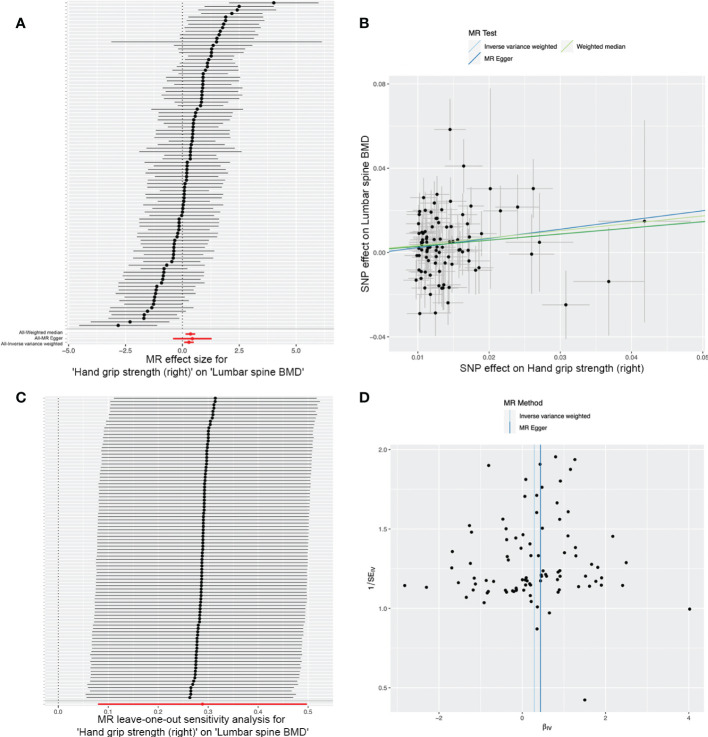
Effects of hand grip strength on lumbar spine BMD. **(A)** Forrest plot. **(B)** Scatter plot. The slopes of each line represent the causal association for each method. **(C)** Leave-one-out analysis. **(D)** Funnel plot.

The MR results between hand grip strength and BMD are shown in **Figure 2**. We selected 97, 92, 93 and 92 SNPs as instrumental variables for the causal analyses between hand grip strength and heel, lumbar spine, forearm and femoral neck BMD, respectively. According to the IVW method, only lumbar spine BMD was casually influenced by hand grip strength (*β*=0.288, 95% confidence interval [CI]=0.079-0.497, *P*=0.007), suggesting that a one-standard deviation (SD, 11.2 kg) increase in hand grip strength was associated with a 0.288-SD increase in lumbar BMD. This result was supported by weighted median sensitivity analyses (*β*=0.347, 95% CI=0.100-0.595, *P*=0.006). There was no evidence of directional pleiotropy among the SNPs associated with hand grip strength in the MR–Egger regression (intercept=-0.002, *P*=0.74). In the leave-one-out analyses, no single SNP strongly drove the overall effect of hand grip strength on lumbar spine BMD. The symmetry in the funnel plots also suggested that there were no violations of the MR assumptions (**Figure 3**). However, no statistically significant relationships between hand grip strength and BMD in the other three skeleton sites (heel, forearm and femoral neck) were observed from the IVW method. The intercepts of the MR–Egger method were 0.001, 0.004 and 0.005, and *P* values for pleiotropy were 0.81, 0.64 and 0.41, respectively, suggesting that there was no directional pleiotropy among the SNPs we used.

### 3.2 Casual relationships between hand grip strength and fracture risk

**Figure 4:**
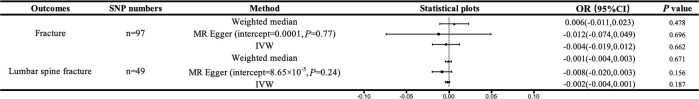
Casual associations between hand grip strength and fracture. IVW, inverse variance weighted; MR Egger, mendelian randomization egger; CI, confidence interval; BMD, bone mineral density; SNP, single nucleotide polymorphism.

The MR results between hand grip strength and fracture risk are shown in **Figure 4**. We selected 97 and 49 SNPs as instrumental variables for the causal analyses between hand grip strength and overall fracture risk and lumbar spine fracture risk, respectively. However, the IVW methods yielded no evidence to support a causal association between hand grip strength and overall fracture risk (*β*=-0.004, 95% CI=-0.0190-0.012, *P*=0.662) or lumbar spine fracture risk (*β*=-0.002, 95% CI=-0.004-0.001, *P*=0.187). No evidence of causal relationship was apparent using the weighted median and MR–Egger methods.

The intercepts of the MR–Egger test were 0.0001 and 8.65×10^-6^, respectively, and the *P* values for pleiotropy were 0.77 and 0.24, respectively, suggesting that there was no directional pleiotropy among the SNPs we used.

## 4 Discussion

The present study aimed to explore whether and to what extent hand grip strength affects BMD and fracture risk. We used GWAS data and performed a state-of-the-art two-sample Mendelian randomization analysis to investigate the causal relationship between hand grip strength and BMD at different skeleton sites and fracture risks. Our results suggested that there was a positive causal relationship between hand grip strength and lumbar spine BMD, which is the most common site of osteoporotic fracture (21), but no causal relationship was found between hand grip strength and BMD at the heel, forearm or femoral neck. However, we found no evidence to support a causal relationship between hand grip strength and fracture risks.

Hand grip strength is a well-established indicator of muscle strength and is the most commonly used measurement in large epidemiological studies to assess muscle condition (22–24). It is a sensitive index for metabolic health, including metabolic syndrome and sarcopenic obesity in the elderly (25, 26). Our previous MR study assessed the causal relationships of overall and central obesity with BMD. In terms of overall obesity, we found that BMI, a measurement of overall obesity, was causally and positively associated with BMD, and the genetic determination of BMI is different but similar across different skeletons (27). In terms of central obesity, our study suggested variations in the ability of different central obesity traits to influence BMD and found that hip circumference adjusted by BMI (negatively) and waist-to-hip ratio (positively) may be important factors causally influencing BMD (28). Recent studies have demonstrated that sarcopenic obesity is associated with an increased risk of physical disability, osteoporosis and nonvertebral fractures in older adults when compared to those with obesity (29, 30). The analysis of body components also revealed that lean mass actually contributes more to BMD than fat mass (31), and whether large BMI is a stronger contributor to lean or fat mass remains unclear (31, 32). Therefore, understanding the hand grip strength-osteoporosis relationship is an important part of obesity-osteoporosis studies, and the present study is an extension of our previous studies. The similarity between this study and our previous MR studies is that they both sought to elucidate the relationship between obesity and osteoporosis using a novel causal arguing method, and examine differences in genetic determinants of BMD measurements between various traits. The novelty of this study is that the use of grip strength as a proxy for sarcopenia provides a more specific analysis of the effect of sarcopenic obesity on BMD from the perspective of genetic variation, which is a transition from the traditional concept of obesity to the new one. Our findings may shed light on the level of grip strength metrics to predict the risk of osteoporosis.

The relationship between hand grip strength and osteoporosis is a crucial public health issue, and risk exposure can slowly progress toward disease. However, there have been controversial results about the role of hand grip strength in osteoporosis. Our results were consistent with previous observational studies showing a positive relationship of hand grip strength with BMD at nonadjacent bones. A cross-sectional study of 1850 American participants found that hand grip strength is associated with increased BMD of nonadjacent bones (femoral neck and total lumbar spine) across gender and menopausal statuses (12). A similar protective effect of hand grip strength on nonadjacent bones was also found in a Chile study including 1427 adolescent students (14) and a small Chinese study including 120 postmenopausal women (33). In terms of adjacent bone, Mclean et al. analyzed the Framingham osteoporosis cohort including 1159 participants and found that higher hand grip strength was associated with higher radius bone size and strength but not volumetric BMD (34). The authors speculate that the unaffected BMD may be because larger bone has similar bone mineral content. Similar positive results were also found in the relationship between the cross-sectional area of the hip flexors and quadriceps for hip BMD (35). However, our study did not find a causal relationship between hand grip strength and forearm BMD. In terms of fracture risk, a population-based study of community-dwelling older adults found that sarcopenic obese older men have over 3-fold increased rate of self-reported fractures over 10 years compared to both non-sarcopenic non-obese and obese alone counterparts (30). However, we did not find a causal association between hand grip strength and fracture risk. The observational nature of these studies did not permit the establishment of causality. Their observation was also limited to a relatively small sample size. Additionally, conventional observational studies cannot distinguish unmeasured confounders or quantify the magnitude of this association.

The relationship between muscle strength and BMD is complex and complicated by many factors. The mechanostat theory posits that mechanical strain applied to bone is a determinant of bone remodeling and that bones adapt not only to static forces but also to the dynamic forces created by muscular contractions (36). Lifting weights increases the load on the lumbar spine and thus increases BMD, which will automatically increase grip strength due to holding on to the weights. In addition, MR analysis lies between traditional observational studies and interventional trials and it is important to triangulate evidence from different studies. We would not expect an IV estimate to reflect the effect of current treatment on prognosis. Therefore, our findings cannot simply be interpreted as increasing lumbar spine BMD by increasing grip strength alone. Endocrine factors also interact with bone modeling. Skeletal muscle can act as an endocrine organ to regulate bone anabolism in a nonmechanical manner (37). Skeletal muscle secretes various myokines (e.g., myostatin, IL6, IGF-1, irisin) in an autocrine, paracrine, or endocrine manner to regulate the metabolic activities of bone cells in various ways and ultimately contribute to the pathogenesis of osteoporosis mechanisms (38). Several studies have indicated that sarcopenia and osteoporosis are co-occurring in the elderly (39, 40), the results of these studies or common sense knowledge may be somewhat misleading to the conclusions of this study. The conclusion of this study, that there was a positive causal relationship between HGS and lumbar spine BMD, was not specific to a particular age, such as the elderly, but was based on a large sample of people after the methodological exclusion of the confounding factor of age. The underlying mechanisms of the effect of muscle strength on BMD, including mechanical and metabolic aspects, still need to be further studied in the future.

This study has several strengths. First, MR may minimize confounding factors and reverse causal effects existing in the observational studies. Second, MR lies between observational studies and interventional trials and provides information about public health interventions in cases when randomized controlled trials may not be feasible. Third, the large sample size and robustly associated SNPs give sufficient power to detect causal effects.

There are still some limitations in the present study. First, all individuals in the study are of European descent. MR is dependent on ethnicity, so it may be inaccurate when extending our conclusions to other populations. Second, although we found no evidence of horizontal pleiotropy in several analyses, we have to admit that MR-Egger regression loosens the constraints and reduces the accuracy of the estimates (41), it is impossible to prove the validity of all three MR assumptions. Nevertheless, considering the unique advantages of MR-Egger regression for detecting and adapting to bias arising from unbalanced pleiotropy, we finally employed this method in the standard MR analysis. Third, we used heel eBMD instead of the standard BMD in this study. However, the potential biological characteristics are similar, and the heel eBMD traits were also successfully utilized in previous MR studies (42–44).

In conclusion, our Mendelian randomization study suggested that there was a positive causal relationship between hand grip strength and lumbar spine BMD, which is the most common site of osteoporotic fracture, but no causal relationship was found between hand grip strength and BMD at the heel, forearm or femoral neck. In addition, no statistically significant effects of hand grip strength on fracture risks were observed. These results should be considered in future research and in the development of public health measures and osteoporosis prevention strategies.

## Data availability statement

Publicly available datasets were analyzed in this study. This data can be found here: MR-Base platform.

## Ethics statement

Ethical review and approval was not required for the study on human participants in accordance with the local legislation and institutional requirements. Written informed consent for participation was not required for this study in accordance with the national legislation and the institutional requirements.

## Author contributions

All authors contributed to the article and approved the submitted version. JS and WW Designed and performed the study. TL, SW and JZ collected and analyzed the data. XD interpreted the results. JS and TL wrote the paper.
